# From Professionalism to Professional Identity: The Emerging Core of Modern Medical Education

**DOI:** 10.12669/pjms.41.12.14174

**Published:** 2025-12

**Authors:** Shabih H Zaidi, Ba’aha Abdulrahman Hadi

**Affiliations:** 1 Shabih H Zaidi, www.sivu.alsadiquni.com UK; 2 Ba’aha Abdulrahman Hadi, Dept. of Medical Education, University of Karbala, Karbala, Iraq

**Keywords:** Professionalism, Professional identity, Medical education, Leadership, Artificial intelligence, Ethics, Professional development

## Abstract

The classical educational triangle—knowledge, skills, and attitude—has long guided the philosophy of medical education. However, the term attitude inadequately reflects the depth and transformative essence of professionalism. This paper argues that professionalism, rather than being a mere behavioural component, embodies the bridge between knowledge and skills through the internalized concept of professional identity. The development of professional identity represents a vital process in shaping authentic, reflective, and ethically grounded physicians capable of navigating contemporary challenges such as digital transformation and artificial intelligence (AI). By embedding professional identity formation as a core educational goal, medical institutions can cultivate resilient, empathetic, and leadership-oriented professionals ready for the future of healthcare.

## INTRODUCTION

The famous triangle of education consists of knowledge, skills, and attitude. We have a strong objection to the nomenclature attitude, as it diminishes the third and most fundamental bedrock of this triangle. It fails to give due status to the bridge linking knowledge with skills—without which the two will run parallel, akin to railway tracks. That junction is better defined as professionalism, which is not merely an attitude or behaviour but a deeper, more comprehensive concept representing professional identity.

## DISCUSSION

There has been increasing discourse on professionalism in recent years, particularly following the extensive work of Edmund Pellegrino,^1^ who emphasized its role in shaping a professional into a recognizable moral and intellectual entity. Yet, the discussion must now evolve toward the essence of professionalism—professional identity.

Cultivating professional identity is an essential developmental step, carrying equal importance to the acquisition of clinical knowledge and technical skills. Professional identity refers to a fundamental representation of the self, achieved in stages over time, resulting in an individual who genuinely “thinks, feels, and acts like a physician”.^2^

The formation of professional identity is the foundational process one undergoes during the transformation from a layperson to a professional. It involves the construction, deconstruction, and assimilation of professional beliefs, norms, values, and behaviours into a pre-existing personal identity or concept of personhood. This intricate process captures how an individual transforms both personally and professionally. When successful, professional behaviour becomes internalized, springing from within rather than imposed externally.

Professional identity thus forms the necessary foundation of professionalism. Both are intimately related and mutually reinforcing, though often treated separately. The process of professional identity development requires integration of the professional self with the personal self. The growing importance of this concept highlights the need for educators to embed it systematically and strategically within curricula.

Modern educational programmes increasingly integrate professional identity formation with leadership development. Leadership capability is essential for future professionals to influence and manage change effectively within evolving healthcare systems. Core competencies such as professional identity, professionalism, leadership, and resilience are now assessed in parallel. Moreover, as digital transformation reshapes the healthcare landscape and artificial intelligence (AI) permeates clinical practice, the cultivation of soft skills such as empathy, reflection, and ethical leadership becomes indispensable for those working alongside technologies like generative AI.

By positioning professional identity formation at the heart of professional development, educators can nurture authentic and resilient leaders who embody the soul of their profession. These professionals are distinguished not merely by external behaviour or superficial attitude but by an internalized, enduring commitment to their professional ethos.

## CONCLUSION

As AI continues to advance within healthcare, each day brings new ethical challenges and responsibilities concerning its application in patient care. Addressing these challenges requires physicians who possess the traits of a well-developed professional identity, achieved through the triple loops of learning—understanding, reflecting, and transforming.^3^

As medical education progresses, the acquisition of professional identity becomes imperative. Only then can physicians authoritatively harness evolving technologies like AI. A thoughtful man–machine symbiosis, grounded in professionalism and guided by a deeply rooted identity, holds the potential to transform patient care in unprecedented ways.
